# Integrated Optical Filters with Hyperbolic Metamaterials

**DOI:** 10.3390/nano13040759

**Published:** 2023-02-17

**Authors:** Mas-ud A. Abdulkareem, Fernando López-Rayón, Citlalli T. Sosa-Sánchez, Ramsés E. Bautista González, Maximino L. Arroyo Carrasco, Marycarmen Peña-Gomar, Victor Coello, Ricardo Téllez-Limón

**Affiliations:** 1 Facultad de Ciencias Físico Matemáticas, Universidad Michoacana de San Nicolás de Hidalgo, Avenida Francisco J. Múgica s/n, Ciudad Universitaria, Morelia C. P. 58030, Michoacán, Mexico; 2 Facultad de Ciencias Físico-Matemáticas, Benemérita Universidad Autónoma de Puebla, Av. San Claudio y 18 Sur, San Manuel, Puebla C. P. 72570, Puebla, Mexico; 3 Centro de Investigación Científica y de Educación Superior de Ensenada, Unidad Monterrey, Alianza Centro 504, PIIT, Apodaca C. P. 66629, Nuevo León, Mexico; 4 School of Biological Sciences, The University of Adelaide, Adelaide, SA 5005, Australia; 5 CONACYT—Centro de Investigación Científica y de Educación Superior de Ensenada, Unidad Monterrey, Alianza Centro 504, PIIT, Apodaca C. P. 66629, Nuevo León, Mexico

**Keywords:** integrated optics, hyperbolic metamaterials, bandpass filter, metaphotonics

## Abstract

The growing development of nanotechnology requires the design of new devices that integrate different functionalities at a reduced scale. For on-chip applications such as optical communications or biosensing, it is necessary to selectively transmit a portion of the electromagnetic spectrum. This function is performed by the so-called band-pass filters. While several plasmonic nanostructures of complex fabrication integrated to optical waveguides have been proposed, hyperbolic metamaterials remain almost unexplored for the design of integrated band-pass filters at optical wavelengths. By making use of the effective medium theory and finite integration technique, in this contribution we numerically study an integrated device consisting of a one-dimensional hyperbolic metamaterial placed on top of a photonic waveguide. The results show that the filling fraction, period, and number of layers modify the spectral response of the device, but not for type II and effective metal metamaterials. For the proposed Au-TiO_2_ multilayered system, the filter operates at a wavelength of 760 nm, spectral bandwidth of 100 nm and transmission efficiency above 40%. The designed devices open new perspectives for the development of integrated band-pass filters of small scale for on-chip integrated optics applications.

## 1. Introduction

Optical bandpass filters are optical devices that selectively transmit a portion of the electromagnetic spectrum while rejecting all other wavelengths. One of the main applications of these devices stands for optical communications, where optical fiber technology requires the transmission of specific bandwidths at given wavelengths. For many years, different photonic waveguides compatible with optical fibers have been designed to properly filter light signals [1,2,3,4,5,6,7]. Even with this development, several factors still hinder the practical use of these devices with current technologies, which require miniaturized functional photonic systems with more advanced and configurable filters with novel characteristics.

With the development of nanotechnology, new opportunities have opened up for the integration of artificially engineered subwavelength materials with enhanced properties not otherwise found in nature, so-called metamaterials [8,9,10], with photonic waveguides. Among the different structures integrated to waveguides for signal filtering that can be mentioned are dielectric and plasmonic ring resonators [11,12], gratings [13,14,15], nanodisk [16,17,18] and asymmetric resonators [19,20], nanostructured plasmonic waveguides [21,22], waveguide cladding modulators [23,24,25,26], and photonic crystals [27,28]. In a previous work, we experimentally demonstrated that a gold nanoslab placed on top of an ion-exchanged glass waveguide serves as a stop-band filter of light for a broad bandwidth at near infrared wavelengths [29].

In recent years, a new kind of metamaterials have attracted the interest of the research community due to their unusual anisotropic nature, the so-called hyperbolic metamaterials (HMM) [30,31,32,33,34]. This growing interest is because isotropic materials have a closed isofrequency surface that limits the wavenumber of the electromagnetic field propagating through these media. For HMM, an extreme anisotropy is induced, leading to higher wavenumbers values in a non-closed hyperbolic isofrequency surface [35,36]. One way to introduce this extreme anisotropy is by alternating dielectric and metallic thin layers [32,37,38,39]. For these one-dimensional periodic structures, intrinsic resonant modes arise from coupling of photonic modes and surface plasmon polaritons at the metal-dielectric interfaces, leading to hybrid photonic-plasmonic modes. If the wavelength and spectral bandwidth of these modes are too close, broad band resonances can take place [37,40]. These broad resonances have been used for the design of bulk bandpass filters operating at telecommunications [41], terahertz (THz) [42], and near infrared [43] wavelengths. Integrated band-pass filters have also been proposed at THz frequencies by using a composite of two different-sized tapered HMM waveguide arrays, with each waveguide operating at wide but different absorption and transmission bands [44].

**Figure 1:**
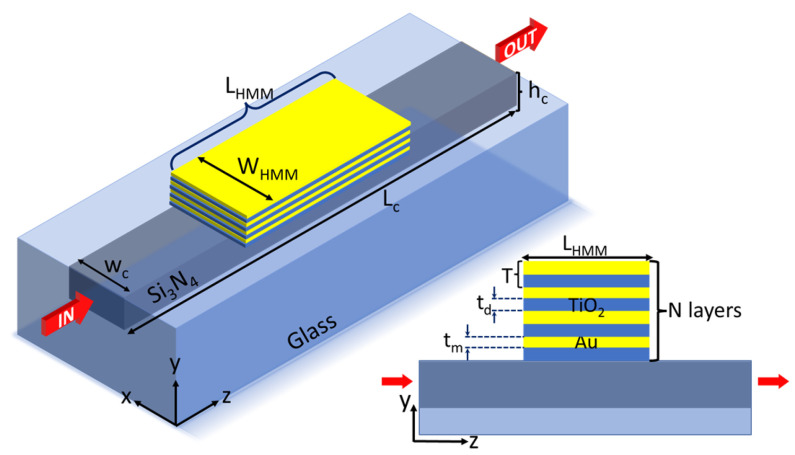
Schematic of the integrated device. Hyperbolic metamaterial consisting of a periodic array of Au-${\mathrm{TiO}}_{2}$ thin layers of thickness $t_{m}$ and $t_{d}$, respectively, are placed on top of a ${\mathrm{Si}}_{3}N_{4}$ waveguide ($w_{c}=750$ nm, $h_{c}=250$ nm) buried in a glass substrate. Photonic modes propagate through the waveguide along the *z* direction from the input (IN) and the transmission spectrum is measured at the output (OUT) face of the waveguide.

However, the use of HMM for the development of band-pass filters integrated to optical waveguides operating at visible and near-infrared wavelengths has barely been explored. These spectral bands are of interest, for instance, for on-chip biosensing applications in the first and second biological windows [45]. In this contribution, we numerically explore the design of an integrated band-pass filter by making use of metallic-dielectric multilayered HMM, The structure, as depicted in Figure 1, consists of a Si_3_N_4_ multimode waveguide on top of which a finite periodic array of gold (Au) and titanium dioxide (${\mathrm{TiO}}_{2}$) thin layers are placed. It is demonstrated that the transmission for the ${\mathrm{TM}}_{0}$ mode is filtered at a central wavelength λ=760 nm of bandwidth $\Delta \lambda_{FWHM}=100$ nm with a transmittance above 40% of incident light, when the multilayered system behaves as an effective metal or hyperbolic metamaterial type II [32], while for an effective dielectric metamaterial, the band-pass filtering can be tuned as a function of the period and number of layers. Due to the simplicity of the structure, the proposed devices open new perspectives for the development of size-reduced integrated optical filters.

## 2. Materials and Methods

### 2.1. Description of the Integrated System

The device under analysis consists of a finite-sized HMM placed on top of a dielectric photonic waveguide, as depicted in Figure 1.

**Figure 2:**
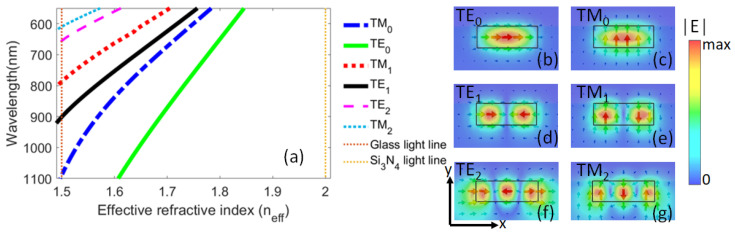
Modes guided by the waveguide. (**a**) Dispersion curves of the dielectric ${\mathrm{Si}}_{3}N_{4}$ waveguide in the spectral range from 500 nm to 1100 nm. The maps show the |E| field distribution and electric field lines, computed at λ=550 nm, of the (**b**) ${\mathrm{TE}}_{0}$, (**c**) ${\mathrm{TM}}_{0}$, (**d**) ${\mathrm{TE}}_{1}$, (**e**) ${\mathrm{TM}}_{1}$, (**f**) ${\mathrm{TE}}_{2}$, and (**g**) ${\mathrm{TM}}_{2}$ modes launched at the input of the waveguide.

The waveguide consists of a rectangular silicon nitride (${\mathrm{Si}}_{3}N_{4}$) core of width $w_{c}=750$ nm, height $h_{c}=250$ nm, and length $L_{c}=4.0$ μm. This core of refractive index $n_{c}=2.016$ was buried in a glass substrate of refractive index $n_{sub}=1.5$. The superstrate was considered as air ($n_{sup}=1.0$). The dispersion curves of the dielectric waveguide and spatial distribution of the electric field of each mode are shown in Figure 2. The modes that can be propagated along the *z* direction of the waveguide in the spectral range from 500 nm to 1523 nm, have cut-off wavelengths $\lambda_{{\mathrm{TE}}_{0}}=1523$ nm, $\lambda_{{\mathrm{TM}}_{0}}=1089$ nm, $\lambda_{{\mathrm{TE}}_{1}}=901$ nm, $\lambda_{{\mathrm{TM}}_{1}}=787$ nm, $\lambda_{{\mathrm{TE}}_{2}}=654$ nm, and $\lambda_{{\mathrm{TM}}_{2}}=611$ nm.

The HMM has a width of $w_{HMM}=1.0$ μm and length $L_{HMM}=2.0$ μm, and it is constituted by a periodic array of *N* alternated thin layers of metal (Au) and dielectric (${\mathrm{TiO}}_{2}$) materials, of thickness $t_{m}$ ad $t_{d}$, respectively. The period of the structure is $T=t_{d}+t_{m}$, as shown in the inset of Figure 1. The HMM on top of the waveguide was centered with respect to the center of the core. The dielectric function of gold was calculated from the Drude–Lorentz model as described in Refs. [46,47], while the refractive index of ${\mathrm{TiO}}_{2}$ was taken from the refractive index database using Ref. [48].

### 2.2. Effective Medium Theory

The effective medium theory describes a system considering the properties of its constituents. For a metamaterial composed of multilayers, if the layers are thinner with respect to the wavelength, it is possible to consider all the multilayers as a whole system whose electrical response can be characterized by an effective permittivity.

**Figure 3:**
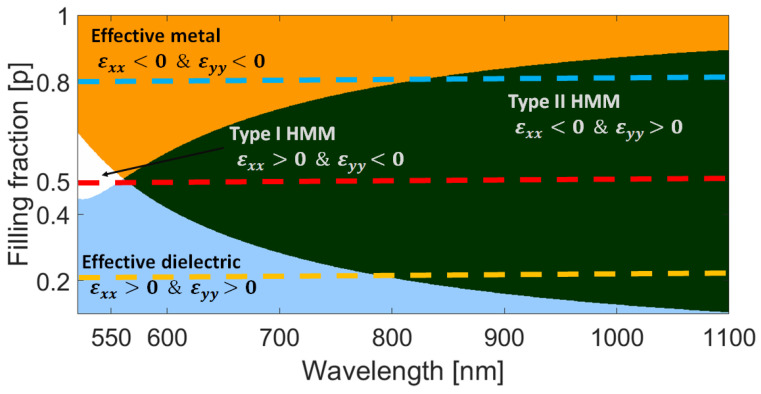
Phase diagram of the metamaterial composed by layers of Au/${\mathrm{TiO}}_{2}$ as a function of the filling fraction and wavelength. The first region in blue (lower left corner) corresponds to an effective dielectric behavior when $\epsilon_{xx}>0$ and $\epsilon_{yy}>0$. The second region in orange (upper left corner) corresponds to an effective metal when $\epsilon_{xx}<0$ and $\epsilon_{yy}<0$. The third region in white corresponds to a Type I HMM for which $\epsilon_{xx}>0$ and $\epsilon_{yy}<0$ and the fourth region in green corresponds to a Type II HMM for which $\epsilon_{xx}<0$ and $\epsilon_{yy}>0$. Dotted lines point out the behavior of the multilayerd system for p=0.2 (yellow), p=0.5 (red) and p=0.8 (blue).

We propose an HMM made by an infinite periodic array of (${\mathrm{TiO}}_{2}$) and gold (Au) thin layers. Considering the constituent materials, filling factor, and using the effective medium theory for a multilayer system [35], the effective permittivity phase diagram (Figure 3) was obtained following the classification of Ref. [32], related to the positive or negative values of the effective permittivity components. If $\varepsilon_{xx}$ and $\varepsilon_{yy}$ have opposite signs, extreme anisotropy is achieved, giving rise to hyperbolic dispersion curves [30,31,32].

The effective permittivity phase diagram (Figure 3) classifies the effective medium according to the effective dielectric permittivity equation, as a function of the metal filling fraction and wavelength. The phase diagram was obtained using the effective medium theory, where the effective dielectric function for transverse magnetic polarization is given by Ref. [35]:

(1) $$\epsilon_{xx}=\epsilon_{zz}=p\epsilon_{m}+\left(1-p\right)\epsilon_{d},$$

(2) $$\epsilon_{yy}={\left(\frac{p}{\epsilon_{m}}+\frac{1-p}{\epsilon_{d}}\right)}^{-1},$$

where $p=t_{m}/T$, is the metal filling fraction (portion of metal at each period). The metallic and dielectric layers have permittivities $\epsilon_{m}$ and $\epsilon_{d}$, respectively. Using Equations (1) and (2), we computed the phase diagram in Figure 3 varying the Au filling fraction from p=0.1 to p=1 for a spectral wavelength range from λ=500 nm to λ=1100 nm. We show different regions depending on the signs of $\epsilon_{xx}$ and $\epsilon_{yy}$. ${\mathrm{TiO}}_{2}$ and Au permittivities were also taken from Ref. [48] and Refs. [46,47], respectively.

### 2.3. Transfer Matrix Method

**Figure 4:**
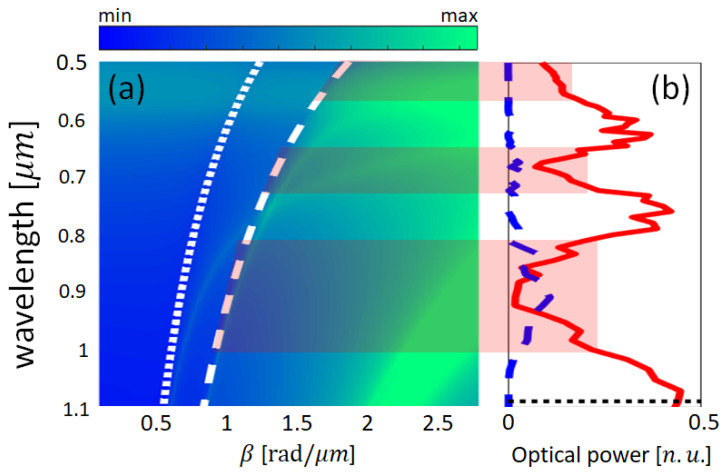
(**a**) Dispersion curves for a HMM of 6 Au and 6 ${\mathrm{TiO}}_{2}$ layers with a filling fraction p=0.5 and period T=80 nm. Dotted and dashed curves represent air and glass light-lines, respectively. (**b**) Normalized transmission (red solid) and reflection (blue dashed) spectra (normalized units) for an integrated system with a finite HMM (N=12, p=0.5, and T=80 nm) integrated on top of a dielectric waveguide. Several modes in the dispersion curves are associated in the main bands corresponding to the broad-band minima in the normalized transmission spectrum (shaded regions).

To compute the dispersion curves of the multilayered media, we used the transfer matrix method [49]. These curves quantify the number of modes supported by the periodic structure as a function of the propagation constant at a given spectral range. The obtained results for a system of N=12 layers (6 Au and 6 ${\mathrm{TiO}}_{2}$ layers) with a filling fraction p=0.5 and period T=80 nm ($t_{m}=t_{d}=40$ nm) are shown in Figure 4a. The green curves represent the modes, the white dotted curve represents the air light-line, and the white dashed curve corresponds to the glass substrate light-line. The map was obtained by equating the fourth element of the general matrix to zero [49] and plotted in logarithmic scale, the maxima values being related to the modes supported by the structure.

### 2.4. Light Propagation in a 3D Integrated Device

To compute the transmission and reflection spectra of light at the output and input of the integrated system, we performed 3D simulations by means of the finite integration technique [50], using the commercial software CST Studio Suite 2020 (Dassault Systems, Vélizy-Villacoublay, France). For this purpose, first we computed the photonic modes supported by the dielectric waveguide and used the spatial distribution of their electromagnetic field and propagated them trough the integrated device. For the simulations, we used a computational window of width $w_{x}=3.0$ μm, height $h_{y}=2.4$ μm, and length $L_{z}=4.0$ μm, surrounded by perfectly matched layers. The transmission and reflection signals were measured defining port monitors at the input and end of the waveguide.

Figure 4b shows the normalized transmission (red line) and reflection (blue line) curves when the integrated device was excited with the fundamental ${\mathrm{TM}}_{0}$ photonic mode. Two main broad minima bands are observed in the transmission spectrum, located at the spectral position of the broad modes supported by the HMM plotted in the dispersion curves.

## 3. Results

We firstly analyzed the dependence of the operation of the device as a function of light polarization. For this purpose, we propagated the fundamental ${\mathrm{TE}}_{0}$ and ${\mathrm{TM}}_{0}$ photonic modes through the waveguide. For the ${\mathrm{TE}}_{0}$ mode, the electric field is mainly oriented along the horizontal *x* direction, while for ${\mathrm{TM}}_{0}$, the electric field is oriented along the vertical *y* direction [20]. For these simulations, we considered a system of N=8 layers (4 Au layers and 4 ${\mathrm{TiO}}_{2}$ layers) with a filling fraction p=0.5 ($t_{m}=40$ nm, $t_{d}=40$ nm, period T=80 nm), on top of the ${\mathrm{Si}}_{3}N_{4}$ dielectric waveguide.

**Figure 5:**
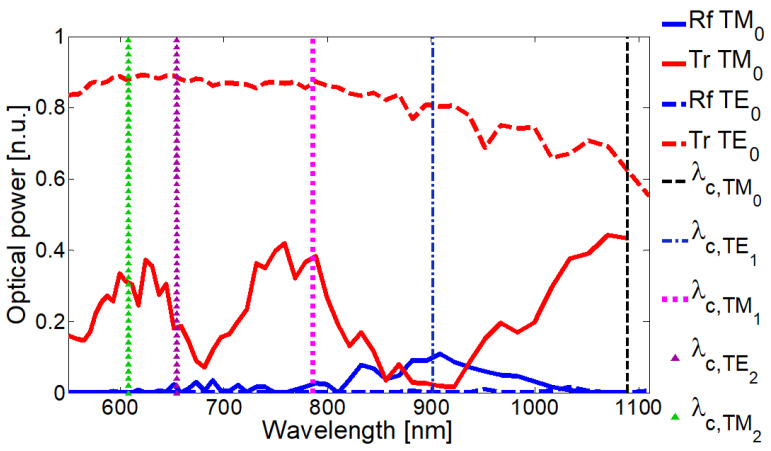
Polarization dependence of transmitted signal. For ${\mathrm{TM}}_{0}$ mode (vertical polarization), the normalized transmission spectrum exhibits two broad deeps due to the excitation of modes in the hyperbolic metamaterial. For ${\mathrm{TE}}_{0}$ mode (horizontal polarization), no deeps are observed as no SPP are excited in the metamaterial.

The results are plotted in Figure 5, where normalized transmission and reflection curves for ${\mathrm{TM}}_{0}$ mode (red and blue continuous, respectively) and for the ${\mathrm{TE}}_{0}$ mode (red and blue dashed, respectively) are shown. Vertical lines correspond to the cut-off wavelengths for each mode supported by the waveguide in the spectral region from 550 to 1150 nm: ${\mathrm{TM}}_{0}$ mode has a cut-off wavelength $\lambda_{c,{\mathrm{TM}}_{0}}=1089$ nm (black dashed), ${\mathrm{TE}}_{1}$ mode at $\lambda_{c,{\mathrm{TE}}_{1}}=901$ nm (blue dot-dashed), ${\mathrm{TM}}_{1}$ mode at $\lambda_{c,{\mathrm{TM}}_{1}}=786$ nm (magenta dotted), ${\mathrm{TE}}_{2}$ at $\lambda_{c,{\mathrm{TE}}_{2}}=655$ nm (purple triangles), and ${\mathrm{TM}}_{2}$ at $\lambda_{c,{\mathrm{TM}}_{2}}=608$ nm (green triangles). We must remark that no mode conversion was observed, and scattering losses are around 10% of incident light: the signal reduction for the ${\mathrm{TM}}_{0}$ mode is mainly because of the optical losses by absorption.

As observed in Figure 5, the transmission of the ${\mathrm{TM}}_{0}$ mode presents the two main broadband deeps centered around 680 nm ($\Delta \lambda_{FWHM}=60$ nm) and at 900 nm ($\Delta \lambda_{FWHM}=150$ nm). For the ${\mathrm{TE}}_{0}$ mode, these broad deeps disappear.

**Figure 6:**
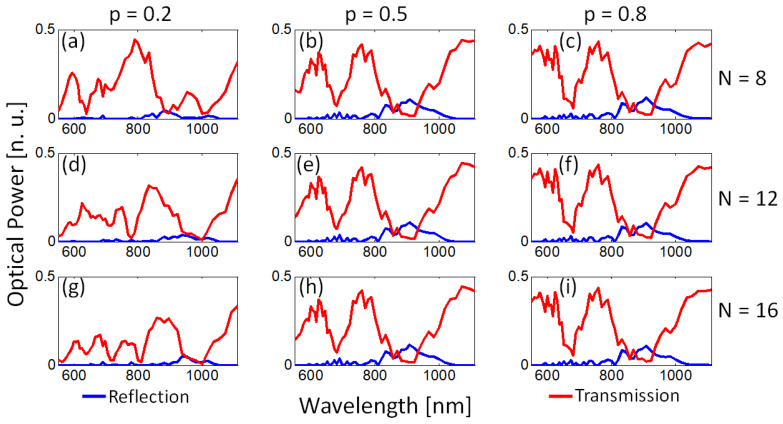
Dependence of the broad deeps as a function of the number of layers (*N*) and filling fraction (*p*). (**a**–**c**) N=8, (**d**–**f**) N=12, and (**g**–**i**) N=16 for p=[0.2,0.5,0.8], respectively. For p=0.2, the number of deeps and their spectral position depends on the number of layers. For p=0.5,0.8, the broad deeps remain almost the same.

As the transmission signal was modified only for ${\mathrm{TM}}_{0}$ mode (vertical polarization), we studied the behavior of the transmission and reflection spectra in terms of the filling fraction, the number of layers, and the period of the structure. We first considered a fixed period T=80 nm for three filling fractions p=[0.2,0.5,0.8] and three values for the number of layers N=[8,12,16] (4, 6, and 8 pairs of Au-${\mathrm{TiO}}_{2}$ interfaces). The results are shown in Figure 6.

The principal observations from transmission (red curves) and reflection (blue curves) spectra of Figure 6 are as follows. For N=8 (Figure 6a–c), the transmission spectrum for p=0.2 exhibits minima at λ=638 nm (guided light transmittance of 2%), λ=880 nm (transmittance of 4%) and λ=999 nm (transmittance of 2%). For p=0.5, two main broad-band deeps occur at 680 nm (transmittance of 7%, $\Delta \lambda_{FWHM}=80$ nm) and at λ=908 nm (transmittance of 1%, $\Delta \lambda_{FWHM}=200$ nm). For p=0.8, two main broadband deeps also appear, centered at λ=680 nm (transmittance of 5%, $\Delta \lambda_{FWHM}=78$ nm) and at λ=908 nm (transmittance of 2%, $\Delta \lambda_{FWHM}=190$ nm). These two deeps generate a band-pass filter with a central wavelength around λ=760 nm, $\Delta \lambda_{FWHM}=100$ nm, and signal transmittance of 41%.

For N=12 (Figure 6d–f), when p=0.2, two local minima occur at λ=778 nm (transmission of 2%, $\Delta \lambda_{FWHM}=35$ nm) and at λ=999 nm (0.9% transmittance, $\Delta \lambda_{FWHM}=130$ nm) and a transparency band is observed centered at λ=845 nm (30% transmittance, $\Delta \lambda_{FWHM}=110$ nm). For p=0.5 and p=0.8, the transmission spectra are almost the same as for N=8.

For N=16 (Figure 6g–i), if p=0.2, four minima are observed centered at λ=638 nm (6% transmittance, $\Delta \lambda_{FWHM}=20$ nm), λ=713 nm (2% transmittance, $\Delta \lambda_{FWHM}=40$ nm), λ=810 nm (2% transmittance, $\Delta \lambda_{FWHM}=30$ nm), and λ=999 nm (0.6% transmittance, $\Delta \lambda_{FWHM}=100$ nm). For p=0.5 and p=0.8, the transmission spectra remain, and, once again, are almost the same as for N=8 and N=12.

**Figure 7:**
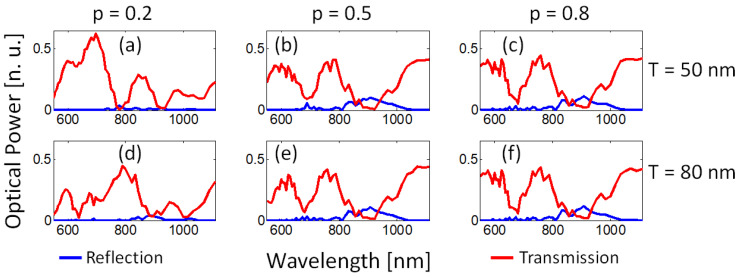
Dependence of broad-band deeps as a function of the period (*T*) and filling fraction (*p*) for a fixed number of layers (N=8 layers). (**a**–**c**) T=50 nm, and (**d**–**f**) T=80 nm, for p=[0.2,0.5,0.8], respectively. For p=0.2 (**a**,**d**), transmission (red solid) and reflection (blue dashed) spectra are modified, while for p=0.5 and p=0.8, they remain almost unchanged.

We then computed the propagation of the ${\mathrm{TM}}_{0}$ mode considering a fixed number of layers N=8 (4 pairs of Au-${\mathrm{TiO}}_{2}$ interfaces) for filling fractions p=[0.2,0.5,0.8] and two periods of the layers T=[50,80] nm. The obtained transmission (red curves) and reflection (blue curves) spectra are shown in Figure 7.

For T=50 nm (Figure 7a–c), when p=0.2 two principal minima occur at λ=778 nm (0.5% transmittance, $\Delta \lambda_{FWHM}=57$ nm) and at λ=936 nm (1% transmittance, $\Delta \lambda_{FWHM}=47$ nm). For p=0.5, two broadband deeps appear centered at λ=689 nm (9% transmittance, $\Delta \lambda_{FWHM}=75$ nm) and at λ=920 nm (0.9% transmittance, $\Delta \lambda_{FWHM}=170$ nm). For p=0.8, two deeps appear centered at λ=680 nm (5% transmittance, $\Delta \lambda_{FWHM}=65$ nm) and at 908 nm (2% transmittance, $\Delta \lambda_{FWHM}=150$ nm). For T=80 nm, the spectra and values are the same as in Figure 6a–c.

## 4. Discussion

The obtained results show that the transmission spectrum of a dielectric waveguide can be filtered by placing a hyperbolic metamaterial consisting of periodically structured metallic (Au)-dielectric (${\mathrm{TiO}}_{2}$) thin layers integrated on top of a dielectric (${\mathrm{Si}}_{3}N_{4}$) waveguide.

This optical integrated filter only operates if light is mainly polarized along the vertical *y* direction, a situation that can be achieved by propagating the ${\mathrm{TM}}_{0}$ mode of the photonic waveguide, as demonstrated in Figure 5. For this polarization, the electric field is symmetrically compatible for the excitation of surface plasmon polaritons at the dielectric-metallic interfaces [29].

As established by the effective medium theory (Equations (1) and (2)), the filling fraction of the multilayered system determines the behavior of the hyperbolic metamaterial (effective dielectric, effective metal of hyperbolic metamaterial types I or II), as shown in Figure 3. Hence, it is expected that the bands of modes supported by the hyperbolic metamaterial (see Figure 4, for instance) also depend on the number of layers (*N*). However, as demonstrated in Figure 6, the number of layers only modifies the spectral response (broadband shifting) of the integrated device when the metamaterial behaves as an effective dielectric material (p=0.2). When the multilayered system behaves as hyperbolic media type II or as effective metal, the number of layers does not significantly modify the central wavelength of two principal broadband resonances centered around λ=680 nm and λ=908 nm, with a transmittance of 5% and 2%, respectively.

When the period of the multilayered structure was modified from T=80 nm to T=50 nm, it was also observed that for p=0.2, different broad-band transmission minima arise and are spectrally shifted, while for p=0.5 and p=0.8 the two main broad-band deeps remain almost unchanged.

It is worth to mention that several small and narrow deeps also appear in transmission spectra. Most of them are due to plasmonic and hybrid photonic-plasmonic modes, which are hard to identify because the modes of the infinite multilayered system are too close to each other (see Figure 4a for instance). In addition, it is possible that some of these small deeps arise from photonic modes, because the hyperbolic metamaterial placed on top of the dielectric waveguide is finite and standing waves can also take place. However, for p=0.5 and p=0.8, these perturbations are mounted in two main broadband deeps.

Even when the proposed structure does not present a high transmission efficiency, multilayered media on top of dielectric waveguides are easier to fabricate. For instance, photolithography combined with the thin layers deposition techniques, such as atomic layer deposition, sputtering, and even thermal evaporation, can be employed with high repeatability, being advantageous in comparison with plasmonic nanostructures of complex geometries. These results open up new perspectives in the design of optical integrated filters by making use of 1D hyperbolic metamaterials. Without the loss of generality, the combination of dielectric and metallic thin layers can be modified to tune the central wavelengths of the proposed integrated band-pass filters.

## Data Availability

The data presented in this study are available on request from the corresponding author.

## Abbreviations

The following abbreviations are used in this manuscript:

TM	Transverse Magnetic
TE	Transverse Electric
Δλ	Wavelength spectral bandwidth
FWHM	Full width at half maximum
